# Out of distribution learning in bioinformatics: advancements and challenges

**DOI:** 10.1093/bib/bbaf294

**Published:** 2025-06-27

**Authors:** Yu Shi, Wei Xu, Pingzhao Hu

**Affiliations:** Biostatistics Division, Dalla Lana School of Public Health, University of Toronto, 155 College Street, Toronto, ON M5T 3M7, Canada; Biostatistics Division, Dalla Lana School of Public Health, University of Toronto, 155 College Street, Toronto, ON M5T 3M7, Canada; Biostatistics Department, Princess Margaret Cancer Center, 610 University Ave, Toronto, ON M5G 2C4, Canada; Biostatistics Division, Dalla Lana School of Public Health, University of Toronto, 155 College Street, Toronto, ON M5T 3M7, Canada; Department of Biochemistry, Western University, 1400 Western Road, London, ON N6G 2V4, Canada; Department of Computer Science, Western University, 1151 Richmond Street, London, ON N6A 3K7, Canada; Department of Oncology, Western University, 800 Commissioners Road, London, ON N6A 5W9, Canada; Department of Epidemiology and Biostatistics, Western University, 1465 Richmond Street, London, ON N6G 2M1, Canada

**Keywords:** out of distribution, transfer learning, domain adaptation, domain generalization, zero-shot learning, foundation model

## Abstract

In the dynamic and complex field of bioinformatics, the development of machine learning models capable of accurately predicting and interpreting genomic data underpins many critical applications, from disease diagnosis to drug discovery. Traditional machine learning models, however, often fail when facing with out-of-distribution (OOD) samples that deviate from their training data, leading to significant performance degradation. This review paper delves into the realm of OOD learning within bioinformatics, highlighting its crucial role in enhancing model generalization and reliability across varied genomic datasets. We provide a comprehensive overview of recent advancements in OOD learning applications, detection techniques, and the integration of foundation models. The discussion extends to various bioinformatics sub-disciplines, including drug discovery, single cell genomics, and polygenic risk score analysis, underscoring how OOD learning has facilitated notable breakthroughs in these areas. Through detailed examination of different model architectures and methods designed to address distribution shifts, we explore the potential of OOD learning to overcome the inherent limitations of standard machine learning models in bioinformatics. This review paper can be served as a valuable resource for bioinformatics researchers, offering a detailed exploration of OOD learning’s transformative impact on understanding complex genomic data and its implications for human health.

## Introduction

In the rapidly evolving field of bioinformatics, the capability to make precise predictions and derive insights from various data is important. Most existing machine learning models are trained based on the assumptions where the test data is assumed to be independent and identically distributed (i.i.d.) from the same distribution as the training data, known as in-distribution (ID) [1]. However, the test samples can be out of distribution (OOD) which results in distributional shift [2]. OOD learning refers to the capability of models to generalize and perform reliably on data that significantly differs from the data used for training [3]. OOD learning is critical in bioinformatics due to the variability and complexity of genomic data. Genomic datasets are often high-dimensional, with intricate patterns that standard machine learning models, trained on limited or specific types of data, struggle to generalize to different domains [4, 5]. The need for robust OOD learning algorithms is underscored by the critical applications of bioinformatics in areas such as disease diagnosis, drug discovery, and understanding of genomic disorders.

This review summarizes the recent developments of OOD learning applications in bioinformatics and related topics including OOD detection and foundation models in bioinformatics. Traditional machine learning models, trained on specific datasets, often fail to maintain their performance when exposed to new, unseen data that deviate from the training distribution [6, 7]. Such weakness can have significant impact in bioinformatics, where the accuracy and reliability of predictions can directly influence human health and disease understanding. The review spans various sub-disciplines, including drug discovery, single cell genomics, and polygenic risk score (PRS). It also illustrates how OOD learning can lead to significant breakthroughs in understanding and predicting related problems. We conducted a review of OOD learning studies in bioinformatics, sourcing articles from PubMed and Google Scholar. This combined search yielded 46 articles and summary of these articles is shown in supplementary information Table S1.

We begin by exploring the OOD learning methodologies based on different model architectures, detailing the different methods tailored to various types of distribution shifts. Subsequently, the review examines several instances where OOD learning has been successfully implemented in bioinformatics. These advancements encompass the development of new algorithms to bolster model robustness, effective integration of diverse data sources, and strategies for enhancing the predictive performance of OOD models. Additionally, we discuss topics related to OOD learning, including insightful thinking on handling distribution shift problems. We conclude by discussing future directions for OOD learning in bioinformatics, including employing foundation models such as GPT-4 to domain specific questions. Despite these advancements, numerous challenges persist. This review also critically examines these challenges, such as the limitations in current datasets, the difficulty in modeling realistic OOD scenarios, and concerns in clinical implementations.

With this comprehensive review, we aim to provide a valuable resource for researchers in bioinformatics and related fields, offering insights into the OOD learning and its transformative potential for understanding and addressing complex bioinformatics problems.

## Distribution shift

### Formalization of distribution shift

We begin by introducing the notations and definitions used in this review [8].

Definition 1. Let $X$ denote an input space and $Y$ an output space. A domain is characterized by data sampled from a distribution, denoted as:

$$ D={\left({x}_j,{y}_j\right)}_{j=1}^n\sim P\left(x,y\right), $$

where $x\in X\subset{R}^d$, $y\in Y\subset R$ denote the corresponding random variables, and $P\left(x,y\right)$ is the joint distribution of the input samples and output labels. $P(x)$ and $P(y)$ represent the distributions of input samples and output labels, respectively.

 

Definition 2. We denote *tr* as the number of training (source) domains, expressed as:

$$ {D}_{train}=\left\{{D}^i|i=1,\dots, tr\right\}, $$

where ${D}^i={\left({x}_j^i,{y}_j^i\right)}_{j=1}^{n_i}$ represents the *i*th domain and ${n}_i$ is the sample size of source domain *i*.

A test domain is defined as,


$$ {D}_{test}=\left\{{D}^k|k= tr+1,\dots \right\}. $$

The joint distributions between each pair of domains are distinct:

${P}^{i_1}\left(x,y\right)\ne{P}^{i_2}\left(x,y\right)$ , $1\le{i}_1\ne{i}_2\le tr$.

The objective of OOD learning is to learn a predictive function from X to Y that is robust and generalizable across training domains to a test domain, where ${P}^k\left(x,y\right)\ne{P}^i\left(x,y\right)$ for any $i\in \left\{1,\dots, tr\right\}$ and $k\in \left\{ tr+1,\dots \right\}$.

### Taxonomy of distribution shift

Traditional machine learning approaches typically assume that both training and test datasets are derived from an i.i.d. environment. However, in real-world applications, this i.i.d. assumption frequently fails due to unforeseen distributional shifts, leading to a significant reduction in model performance upon implementation [9]. Formally, the joint distribution can be factorized in two ways: $P\left(x,y\right)=P(x)P\left(y|x\right)$ and $P\left(x,y\right)=P(y)P\left(x|y\right)$. Such distributional shifts can occur due to covariate shift, label shift, and concept shift [10, 11].

We define two domains as distinct if they differ in at least one of their constituent components, such as $P(x)$, $P\left(y|x\right)$, $P\left(x|y\right)$, $P(y)$, or the joint distribution $P\left(x,y\right)$. Consider a scenario where we aim to estimate a function from $X$ to $Y$ or a conditional distribution $P\left(y|x\right)$ in a causal direction, implying that $X$ is the cause and $Y$ is the effect. Given training points from $P\left(x,y\right)$ and an additional set of inputs sampled from ${P}^{test}(x)$ with $P(x)\ne{P}^{test}(x)$, it is not necessarily true that a change in $P(x)$ leads to a change in $P\left(y|x\right)$ by independence of mechanism and input. Hence, we conclude,


$$ {P}^{test}\left(y|x\right)=P\left(y|x\right). $$

This situation is known as covariate shift [12]. In contrast, concept shift occurs when $P(x)$ remains constant, but the relationship between input data and output variables ($P\left(y|x\right)$) changes over time. This distinction is crucial, as concept shift implies a change in the relationship between inputs and outputs, unlike covariate shift, which involves only a change in the input data distribution.

We now consider the anti-causal prediction direction, where the effect is used as the input to predict the cause variable. In this scenario, $P\left(x|y\right)$ represents the causal mechanism generating X from Y, and it is independent of the cause’s distribution $P(y)$. Label shift occurs when $P(y)$ changes but $P\left(x|y\right)$ remains constant.

OOD generalization algorithms are designed to achieve effective generalization performance under unknown distribution shifts. This area has attracted increasing attentions within the research community, driven by the growing need to effectively handle unobserved data [13]. By combining the strengths of machine learning with the principles of OOD generalization, these algorithms strengthen models to perform reliably on data with distribution shift.

## Taxonomy of OOD learning in bioinformatics

Extensive research has been conducted on addressing domain shift in bioinformatics, as documented in the literature. This has prompted the development of various research topics centered around generalization. In our efforts to tackle domain shift, we have identified four principal categories of analytic methods.

OOD learning enables computational models to effectively handle data that differs from their training distribution. This approach has significantly enhanced machine learning performance across various domains. In computer vision, it has led to more robust image recognition and analysis systems [14]. In the field of natural language processing (NLP), OOD learning has improved the adaptability of models to diverse semantic data [15]. Furthermore, in medical and bioinformatics domains, OOD learning has shown impressive results, such as in predicting patient outcomes based on biological data, identifying novel biomarkers for disease diagnostics or drug discovery. Publicly accessible computation tools for bioinformatics application are presented in Table S2.

### Transfer learning in bioinformatics

**Figure 1:**
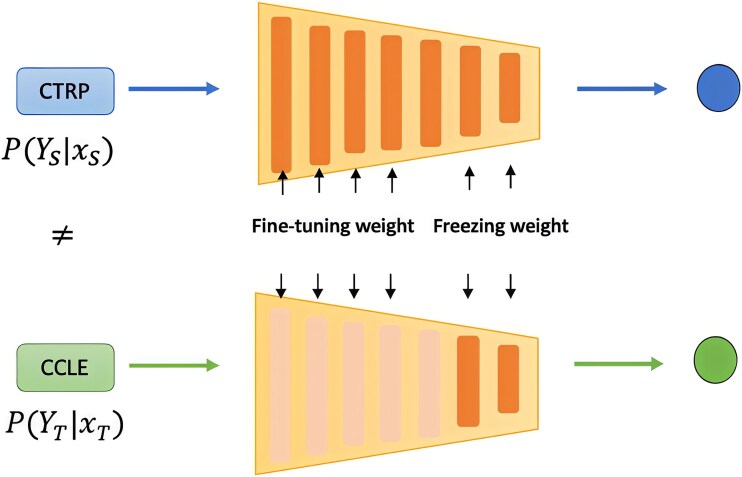
Example of transfer learning scheme. The source data utilizes the in vitro drug screening dataset from the Cancer Therapeutics Response Portal v2 (CTRP), while the target data employs the Cancer Cell Line Encyclopedia (CCLE). The approach trains a prediction model on the source dataset and then refines it on the target dataset. During the transfer learning step, the last two layers are frozen, and the top five hidden layers are fine-tuned.

This method involves leveraging knowledge gained from one task and dataset to enhance performance on a different task and dataset, which may not be closely related to the original. The objective is to reduce learning costs and enhance model performance [16]. Specifically, transfer learning (TL) addresses changes in $P\left(y|x\right)$ in terms of disjoint label space by training a model on a source task to boost its efficacy on a related target task. According to Zhuang et al. [17], TL can be divided into four types: instance-based, feature-based, parameter-based, and relational-based. Instance-based TL employs strategies to weigh instances differently. Feature-based TL alters original features to generate new representations. Parameter-based TL involves transferring model parameters, often by pre-training and fine-tuning process. Relational-based TL focuses on transferring logical relationships from the source to the target domain. Figure 1 illustrates an example of parameter-based TL methods, specifically highlighting the pre-training and fine-tuning scheme, based on the methodology described in [18].

From Cai et al., TL is extensively applied to drug discovery [19]. Hu et al. demonstrated the efficacy of pretraining of graph neural networks (GNNs) on auxiliary data sets, resulting in an average increase of 7.2% in the area under the Receiver Operating Characteristic curve (ROC-AUC) for molecular property prediction tasks [20]. Similarly, Li et al. proposed a long short-term memory (LSTM) model initially trained on one million unlabeled molecules, then fine-tuned for various Quantitative Structure–Property Relationship (QSPR) and Quantitative Structure–Activity Relationship (QSAR) tasks [21]. Evaluation of their models achieved comparable or superior prediction accuracy. Furthermore, Awale et al. trained an LSTM using molecules from data sets including ChEMBL, DrugBank [22], subsequently fine-tuning it to generate analogs of the drug [23]. To enhance results for specific targets in Visual Screening (VS), Imrie et al. fine-tuned a universal model for particular targets, creating protein family-specific models and yielding average improvements of 18.3–24.0% in terms of the area under the precision-recall curve (AUC-PRC) value [24].

Beyond drug discovery, further research in drug analysis also emphasizes the effectiveness of TL. In a study by Zhu et al., Deep Neural Network (DNN) models were employed to illustrate how ensemble TL can enhance drug response prediction (DRP) [18]. This research utilized extensive datasets, with smaller datasets serving for additional model refinement and testing. In the area of Mechanisms of action (MoA) prediction of drugs, Ferreira et al. investigated TL’s application by initially developing a model for common MoAs and then leveraging it to guide the learning process for rarer MoAs [25]. Meanwhile, Kim et al. demonstrated the advantages of applying TL from data-rich to data-poor tissues, thereby improving the accuracy of drug synergy predictions in both well-researched and underexplored tissues [26].

High-dimensional datasets, such as single-cell RNA sequencing (scRNA-seq), are extensively utilized in bioinformatics for their invaluable insights into cellular heterogeneity and complex biological processes. However, challenges arise due to the data’s high dimensionality, sparsity, and batch effects. TL offers a promising solution to these challenges by leveraging knowledge from pretrained models. Lotfollahi et al. developed Single-Cell Architectural Surgery (scArches), a technique for mapping query datasets onto a reference [27]. This method involves pre-training using public datasets and fine-tuning by adding input nodes and weights for new studies. In another study, Alghamdi et al. employed several pre-trained models and fine-tuned a connected classifier for prediction tasks [28]. The study achieved a high average balanced accuracy (BAC) in examining single-cell gene regulatory network images related to type 2 diabetes. Hu et al. applied TL in scRNA-seq clustering, pre-training models on cells with well-annotated cell type labels and fine-tuning them on cells requiring clustering by transferring weights [29]. Lieberman et al. trained a model on previously labeled data, utilizing informative features for classification with the XGBoost classifier [30]. This research underscored the scalability of TL and its enhanced performance on larger and imbalanced datasets, specifically in the classification of single cells. Finally, Wang et al. introduced a novel approach for removing batch effects in scRNA-seq data using deep autoencoder and TL across multiple batches from various cell populations [31]. This method successfully transferred information among batches, effectively mitigating batch effects even across highly diverse batches.

Recent studies have demonstrated the potential of combining TL with Genome-wide Association Studies (GWAS) and PRS to enhance predictions in understudied populations. In their research, Jeng et al. utilized GWAS summary information from base data as knowledge derived from a pre-trained model [32]. This knowledge was then applied to construct the PRS for target individuals using the more ancestrally diverse base data. Such applications of TL showcase its capability to significantly improve model performance in novel tasks.

### Domain adaptation in bioinformatics

**Figure 2:**
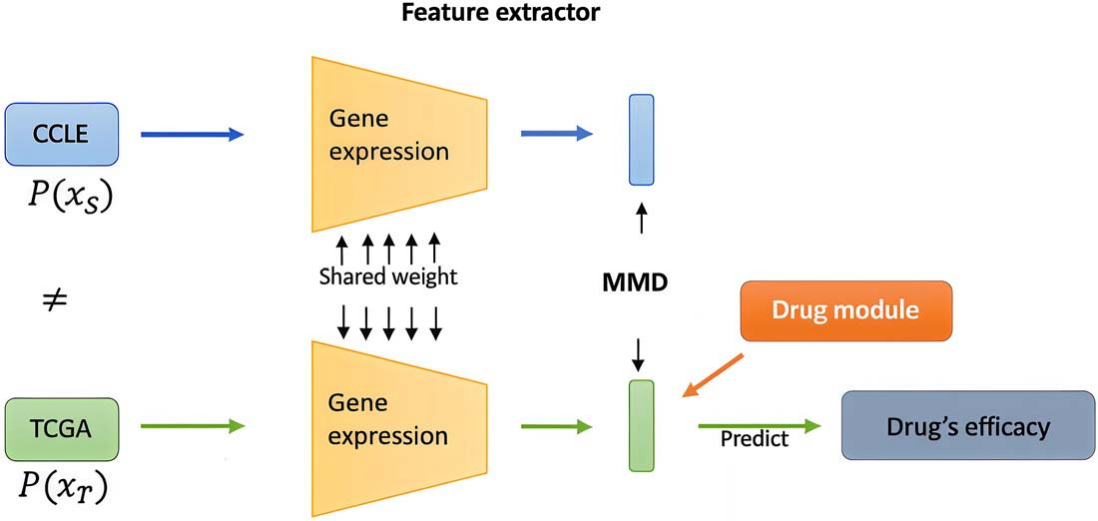
Process of discrepancy-based domain adaptation architecture. Shared weights are employed to extract source expression features from the Cancer Cell Line Encyclopedia (CCLE) and target expression features from The Cancer Genome Atlas (TCGA). To align the representations from both the source and target, Mean Maximum Discrepancy (MMD) is used to minimize the distance between them. Finally, this architecture is utilized to make predictions for the IC50 values.

Recognized as a subset of TL, domain adaptation (DA) is classified under the transductive TL category. DA relies on the presence of sparsely labeled or unlabeled target data to facilitate model adaptation, thereby having access to the marginal ${P}^{test}(x)$ [33, 34]. The main goal of DA is to manage shifts in $P(x)$ between training and testing phases, enhancing the model’s adaptability to new, unseen data. As categorized by [35, 36], shallow DA methods include instance re-weighting, parameter adaptation, feature-based, and landmark selection techniques. Instance re-weighting methods focus on computing the weight of each instance to adjust for domain differences. Parameter adaptation explores various options for adapting classifiers trained on the source domain. Feature-based DA encompasses feature augmentation [37], feature space alignment [38], and feature transformation [39, 40]. Recent advancements in deep convolutional architectures have significantly enhanced performance using DL framework. Deep DA methods based on DL architectures can be divided into three categories based on their methodology [41]. Discrepancy-based methods, inspired by the shallow feature space transformation approaches, typically employ a discrepancy measure calculated between corresponding layers in a Siamese architecture [42]. Adversarial network-based DA employs a generator-discriminator architecture. The generator is tasked with producing domain-invariant and discriminative features from the domains. Deep reconstruction-based DA focuses on creating domain-invariant features from each domain using external reconstruction techniques [43]. Figure 2 displays an example of discrepancy-based DA approach, constructed based on the findings of [44].

DA has been notably employed in DRP studies. Mourragui et al. conducted a DA study to transfer DRPs from preclinical models to human tumors [45]. Their performance comparison revealed a strong association between known biomarkers in skin and breast cancer and relevant drugs. Chen et al. explored the DRP in high-throughput data using a domain-adaptive neural network [46]. The network utilized bulk RNA data as source data to learn and predict drug responses in the target domain, effectively maintaining single-cell heterogeneity while training with both scRNA and bulk RNA data. In another approach, Abbasi et al. utilized graph convolutional network combined with an adversarial DA network to mitigate the distributional difference of compounds between source and target assays [47]. Soufiane et al. employed DA to build a consensus space, leveraging this space to construct robust drug response predictors that transfer effectively from preclinical models to human tumors [48]. Finally, Anastopoulos et al. developed a Mean Maximum Discrepancy (MMD)-based DA model [44]. This model aligns gene expression between cell lines and patient samples of the same tissue of origin, achieving improved discrimination between sensitive and resistant patients compared to the state-of-the-art methods.

Lin et al. applied DA to transfer label knowledge from RNA-labeled data (source domain) to unlabeled scRNA-seq and ATAC-seq data (target domain) for integration of sc-RNA data [49]. This approach proved more effective than previous methods, when dealing with intricate data comprising both biological information and technical variations. Sh et al. introduced the pan-Cancer Seeker (CaSee) to distinguish between normal and cancer cells in scRNA data [50]. The model was trained on 18 types of pan-cancer bulk RNA-seq data using a capsule network combined with DA. It exhibited superior performance against other existing methods, successfully differentiating tissues, cell line sources, and xenograft cells. Genevieve et al. utilized scRNA datasets to define latent spaces in the source domain using mouse retina data and evaluated the latent spaces in target domain using human retina data [51]. This method may help in new cell annotation, cross-species analysis, and linking genomic regulatory and transcriptional signatures. Lopez et al. designed a deep generative model that integrates spatial transcriptomic data with scRNA-seq data, capable of imputing missing genes [52]. This model builds upon DA principles, creating latent spaces refined by the loss of an adversarial classifier. Ren et al. employed a multiple-adversarial domain adaptation network to remove batch effects within the reference dataset, achieving accurate cell type annotation within diverse disease contexts [53]. Similarly, Zhao et al. used a Generative Adversarial Network (GAN) to reduce potential batch bias and enhance batch integration performance [54]. Zhou et al. introduced an adversarial domain adaptation network known as scAdapt [55]. This network is designed to facilitate the transfer of cell labels across datasets impacted by batch effects for classification of single cell. scAdapt has been shown to surpass existing methods in terms of classification performance. Liu et al. focused on extracting common cell type-specific information in a shared feature space across multiple reference datasets, minimizing discrepancies between the ensemble reference and query datasets [56]. For scRNA-seq integration, Sun and Qiu implemented the Siamese Network to develop a shared embedding space, effectively integrating multiple batches [57–59].

DA has also been applied in gene set enrichment analysis. Cai et al. utilized a discrepancy-based DA approach, yielding more robust and focused results compared to other methods, particularly in predicting enriched pathways [60]. In another study, Li et al. employed DA to map both gene-level and isoform-level features into a latent variable space [61]. This strategy allowed for the leveraging of gene domain information in predicting isoform functions, illustrating the versatility of DA in gene-related research.

### Domain generalization in bioinformatics

**Figure 3:**
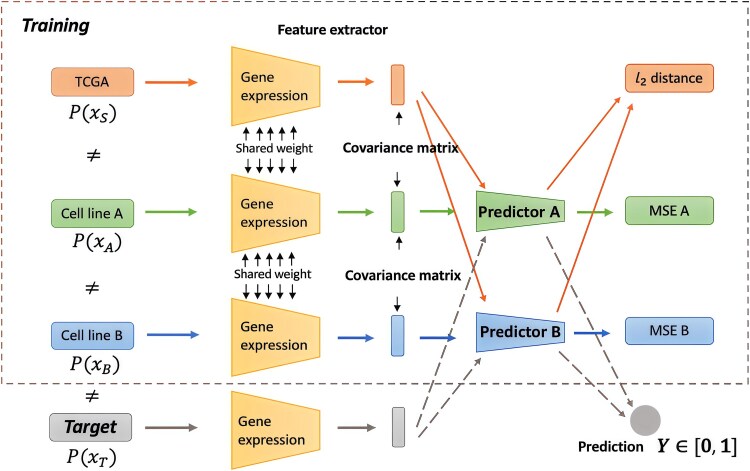
Example of domain generalization architecture. This architecture utilizes a shared feature extractor to process gene expression data from two cell line datasets (CTRPv2 and GDSCv2) and TCGA. The computation of the total loss comprises three components: (i) the supervised loss from both the CTRP and GDSC predictors; (ii) the consistency loss for unlabeled samples, calculated between the predictions from the CTRP predictor and those from the GDSC predictor; and (iii) the alignment loss for any two input domains, achieved by minimizing the difference between their covariance matrices. The architecture enables unseen target domains to extract features and make predictions using the trained model.

In many applications, obtaining or even identifying target data before model deployment is challenging. To handle the domain shift problem when the target data is unavailable, Blanchard et al. introduced the concept of **domain generalization (**DG) [62]. DG focuses on developing a model across one or multiple connected domains, striving for effective generalization to new, unobserved test domains. In DG, distribution shift is primarily attributed to covariate shift [63], under the assumption that the conditional probability $P\left(y|x\right)$ remains stable or changes smoothly relative to the marginal $P(x)$. DG methodologies, as classified by [64, 65], are categorized into three primary approaches: data augmentation, representation learning, and meta-learning strategies. In DG, data augmentation involves manipulating input data to aim in learning general representations that are less sensitive to domain variations. The objective of representation learning is to capture the intrinsic variations within data that remain largely invariant to changes across different domains. Meta-learning strategies focus on learning a general model capable of handling multiple tasks, employing methods such as optimization-based techniques [66], metric-based learning [67], or model-based methods [68]. Figure 3 illustrates an example of DG using representation learning, adapted from [69].

DG has also shown significant promise in drug-related research. Yao et al. developed a model that learns a set of training-domain-specific functions during the training phase and reweights them according to domain relations in the test phase [70]. Applied to molecular-protein interaction prediction, this model demonstrated an average performance improvement of 10.6%. In another approach, Sharifi et al. utilized gene expression data from labeled cell line and patient datasets as input domains to predict drug responses [69]. Their method, employing a shared feature extractor and domain-specific predictors, exhibited substantially enhanced performance across various clinical and preclinical pharmacogenomics datasets. Additionally, Yang et al. treated each patient as an individual domain, addressing the challenge of many-domain generalization [65]. Their method, which integrates mutual reconstruction and orthogonal projection, effectively removes patient-specific covariates to learn label representations. This technique was applied to drug recommendation on the MIMIC-III dataset, where it achieved the best performance. DG has also been effectively applied in single cell data. Pernice et al. and Umer et al. focused on training a robust classifier for white blood cells classification, learning invariant features across distinct domains [71, 72]. Their approach outperformed existing methods, achieving the best F1 macro score and effectively considering rare cell types.

DG’s application extends to clinical prediction as well. For instance, Guo et al. conducted a study to examine the effects of temporal dataset shifts on clinical prediction models [73]. Their research focused on benchmarking DG and DA algorithms to improve the robustness of these models. Pfisterer et al. evaluated the generalization capability of models in clinical survival analysis studies [74]. Furthermore, Aubreville et al. assessed mitotic figure recognition across multiple tumor types from different laboratories [75]. Alongside augmentation techniques, several research teams have implemented specific strategies aimed at domain generalization, including meta-learning approaches.

### Zero-shot learning in bioinformatics

**Figure 4:**
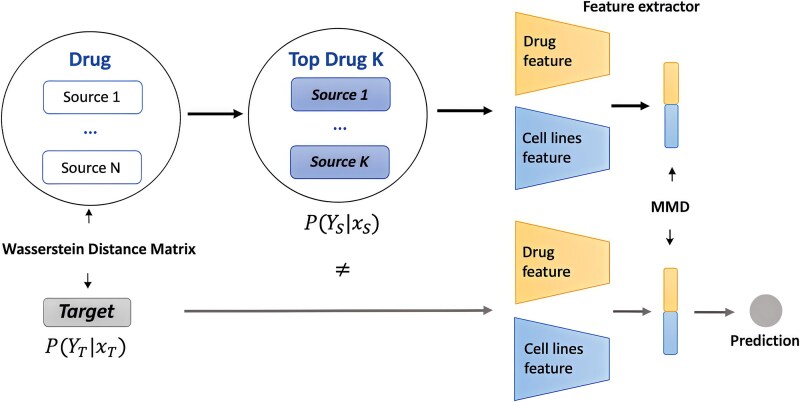
Example of zero-shot learning for unknown compound prediction. This approach begins by selecting K source drug domains that are most similar to the target domain, determined by a Wasserstein distance matrix. It then extracts drug features and cell line features from both source and target domains. The prediction model incorporates a supervised loss from the source domain and a domain adaptation branch for at least one target domain using the MMD method. This structure allows for the prediction of drug responses in the target domain.

The objective of **zero-shot learning (**ZSL) is to enable a model to recognize and understand new, unseen classes during training, using knowledge and patterns it has learned from previously encountered classes. ZSL is used in various applications such as image and speech recognition, NLP, and more. The use of zero-shot learning in bioinformatics is motivated by the challenge of interpreting vast, complex, and often poorly annotated biological data. ZSL techniques are generally applied to various contexts in the field of bioinformatics such as protein function prediction, drug discovery and repurposing and so on. ZSL methods can be broadly categorized into three types: attribute-based methods, embedding-based methods, and generative-based methods [76]. Attribute-based methods train classifiers using labeled features from specific data classes, leveraging these attributes to generalize to unseen classes. Embedding-based methods classify data by measuring the similarity between the semantic embedding of a given sample and the embeddings of potential target classes. Generative-based methods utilize auxiliary information to synthesize sample data, enabling the model to generalize to unseen classes by generating representative data points. Figure 4 presents an example of ZSL, demonstrating the learning of invariant features based on the methodology described in [77].

ZSL in bioinformatics has emerged as a pivotal approach for predicting or classifying biological entities or phenomena not explicitly seen during model training. Wang et al. introduced a protein-specific zero-shot predictor for predicting drug-target interaction, utilizing the meta-learning framework MAML++ in the training phase [78, 79]. The model excelled all baseline methods across transductive, semi-inductive, and inductive test sets. Li et al. proposed a zero-shot learning solution for the DRP in preclinical drug screening [77]. The model integrates with traditional DRP methods, enhancing predictions of unlabeled compounds by learning invariant features from the prior response data of similar drugs. Huang et al. introduced a geometric deep learning approach for zero-shot drug repurposing, facilitating therapeutic predictions for diseases lacking available treatments [80]. The model, trained on a medical knowledge graph, employs a graph neural network and metric-learning module to rank therapeutic candidates. Long et al. separated the drug design process into two phases: sketching and generating [81]. The generating stage leverages a generative pre-trained model to refine the molecular shape obtained in the sketching stage. Leveraging information from databases like DrugBank and PubChem (https://pubchem.ncbi.nlm.nih.gov), Zhu et al. designed a specific prompt for the drug–drug interaction prediction [82]. They fine-tuned a pre-trained language model to encapsulate domain knowledge, followed by reinforcement learning (RL) to generate concise, relevant descriptions for each drug pair. Similarly, Meier et al. demonstrated that language models trained on extensive protein sequence databases can predict protein function measurements without additional supervision [83]. Sivarajkumar et al. also developed a novel prompt-based clinical NLP framework, applying prompt-based learning to clinical texts [84]. Furthermore, to aid zero-shot molecular tasks, Zhao et al. enhanced large language models to encompass both graph and textual data [85]. They implemented transformer mechanisms with generalized position embeddings and decoupled the graph encoding from specific tasks using attention masks. Basu et al. proposed using Siamese Networks to map gene sequence similarities among different COVID-19 variants, utilizing the learned embedding for new variant detection [86]. Kulmanov et al. synergized neural networks with a model-theoretic approach for protein function prediction [87]. This innovative model facilitates zero-shot learning by generating an embedding space from training data corresponding to specific classes, which are then utilized in the axiom. Dong and Kluger presented a model for learning batch and biological patterns form atlas-level scRNA-seq data, treating genes as functions of the latent biological processes [88]. The model addresses sparse shifts due to batch effects and biological changes, using advances in causal representation learning.

**Figure 5:**
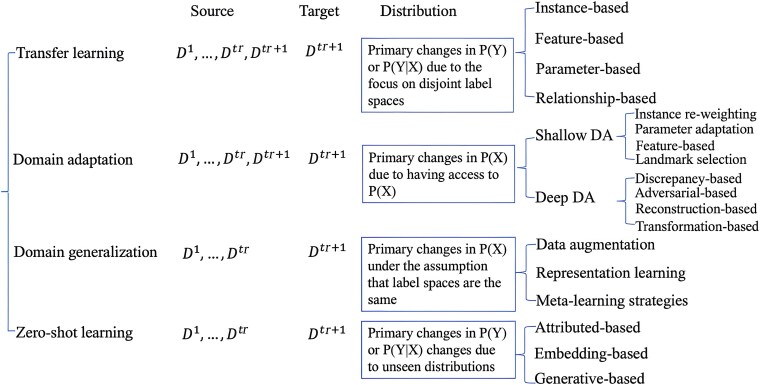
Taxonomy of analytic methods of OOD learning in machine learning. Flowchart outlines four main types of out-of-distribution (OOD) learning: Transfer Learning, Domain Adaptation, Domain Generalization, and Zero-Shot Learning. Each method is defined by the relationship between source and target data and differences in data distribution. Subcategories are listed to show the techniques used within each category.

A summary of these methodological taxonomies is illustrated in Fig. 5.

## O‌OD detection

Identifying distribution shifts is crucial in OOD detection tasks. In such tasks, the range of labels may vary between ID and OOD data, requiring the model to sometimes withhold predictions as suggested by [89]. The primary goal of OOD detection is to recognize test samples that come from a distribution different from the one used in training. Liu et al. note that in cases of major domain shifts, it is often safer to reject major outliers instead of attempting adapted predictions that carry high uncertainty [90]. Several methods and techniques for OOD detection have been developed recently. For example, Lee et al. employed generative models, using GANs to simultaneously train a classifier with an added objective of encouraging low confidence in generated samples [91]. Hendrycks et al. utilized statistical methods, setting a threshold on softmax outputs in DNN to differentiate ID data from OOD data [92]. Additionally, Vyas et al. adopted an ensemble-based approach, training multiple self-supervised classifiers and randomly designating training data as OOD to obtain an OOD detection score [93].

OOD detection is particularly crucial in bioinformatics due to the critical nature of biological data and its significant implications in healthcare and research decisions. OOD detection ensures the reliability and accuracy of predictions from computational models. This is especially important given the high dimensionality and complexity of bioinformatics data, such as genomic sequences or protein structures. Table S3 lists common public data sources used in bioinformatics applications. OOD detection is instrumental in identifying novel genes, proteins, or disease markers not previously observed. In summary, OOD detection plays a pivotal role in ensuring that computational models in bioinformatics are robust, reliable, and relevant to real-world data. It significantly enhances the trustworthiness and efficacy of bioinformatics tools in both research and clinical settings, contributing substantially to advancements in healthcare.

## Training and evaluation strategies

We discuss four methods—transfer learning, domain adaptation, domain generalization, and zero-shot learning—used in OOD learning in bioinformatics. These methods are interrelated yet distinct. Table 1 outlines training strategies for addressing OOD issues, particularly when there is a significant distributional divergence between training and target data. Employing OOD learning strategies is advantageous for bridging this divergence.

**Table 1:** Summary of training strategies

Methods	Strategies	Application	Reference	Data Needed During Training
Transfer Learning	Pre-training and Fine-tuning	Drug discovery, Drug response prediction, Single-cell related application	[18, 20, 21, 23–32, 49]	Source data & Target data
Domain Adaptation	Discrepancy-based, Adversarial-based, Feature-based	Drug discovery, Drug response prediction, Single-cell related application	[44–48, 50, 52–57, 60, 61]	Source data & Target data
Domain Generalization	Representation learning	Drug discovery, Drug response prediction, Single-cell related application, Clinical prediction	[65, 69, 70, 72–75]	Source data
Zero-shot Learning	Prompt-based learning, Meta-learning strategies	Drug discovery, Drug response prediction, Clinical application	[77, 78, 80–88]	Source data

When target data is accessible during training, TL or DA are preferable as these strategies extensively leverage target data. Specifically, TL is suited for scenarios with disjoint label spaces, indicating changes in $P(y)$ or $P\left(y|x\right)$, making it the strategy of choice. Conversely, DA is recommended when no label information for the target data is available, and only the marginal $P(x)$ is accessible and different. In situations where access to target data is not available during training, DG or ZSL are typically selected. ZSL and DG share the objective of addressing unseen distributions. The primary distinction lies in the type of the distribution shift; ZSL primarily deals with shifts in the label space, particularly where ${P}^{test}(y)\ne{P}^{train}(y)$, focusing on the recognition of new and unseen classes. DG, on the other hand, is better suited for scenarios where the training and testing data belong to the same classes but exhibit different marginal distributions, with domain shifts primarily arising from these marginal distribution changes.

Research in this area commonly employs several evaluation metrics to assess the performance of classification datasets. For binary classification tasks, ROC-AUC, the precision-recall area under the curve (PR-AUC), and the F1 score are widely recognized and utilized metrics. For regression datasets, the root mean squared error (RMSE) or mean squared error (MSE) are typically used. Additionally, some studies incorporate the Pearson correlation coefficient and the Spearman correlation coefficient as evaluation metrics. In clustering analysis, the average silhouette width (ASW), local inverse Simpson’s index (LISI), and Adjusted Rand Index (ARI) serve as the metrics for evaluation. To assess a model’s generalization ability, the methodology frequently involves a random split of the dataset into training, validation, and test sets. This division guarantees that the unseen data in the test set thoroughly assesses the model’s generalization capability.

## A case study on OOD learning

In this section, we present a case study highlighting the application of OOD learning within bioinformatics. The model architecture is depicted in Fig. 2. Anastopoulos et al. tackled the inherent differences between cancer cell lines and primary tumors by developing an MMD-based deep learning framework, named PACE, to model patient clinical drug responses [44]. A noticeable variance in RNA-seq profiles was observed between patient data from TCGA and the source cancer cell line (CCL) data. When applying OOD learning to this scenario, the initial step involves assessing the availability of target data, specifically patient information, during the training phase. Given such access, the inclination is towards utilizing TL or DA. However, due to the absence of labels in the TCGA target domain data, the authors opted for DA to effectively align features across both domains. This strategy enabled the prediction of drug responses by leveraging the unlabeled target data, thus bridging the domain gap between the source CCL data and the target TCGA patient data.

The method employed MMD to adjust the latent distribution produced by the expression module, aligning cell line gene expression with patient gene expression. To evaluate the model’s efficiency on patient data, PACE was benchmarked against Tissue-Guided LASSO (TG-LASSO) and noPACE methods in discriminating between resistant and sensitive patients [94]. The noPACE method refers to the strategy where patient data is not incorporated or adapted during the training process. PACE demonstrated a notable improvement in distinguishing resistant and sensitive patients. In the experiment, PACE successfully differentiated nine out of the twelve drugs, in contrast to six by noPACE and three by TG-LASSO, as detailed in Table 2. The results indicate that DA-adapted method yielded a highly informative model, potentially extendable to patient-specific settings.

**Table 2:** Comparison of discrimination ability of PACE, noPACE and TG-LASSO

Drug name	Significant discrimination with PACE	Significant discrimination with noPACE	Significant discrimination with TG-LASSO
bicalutamide	No	No	No
bleomycin	No	No	No
cisplatin	Yes	Yes	Yes
docetaxel	No	No	No
doxorubicin	Yes	Yes	No
etoposide	Yes	Yes	Yes
gemcitabine	Yes	No	No
paclitaxel	Yes	Yes	Yes
sorafenib	Yes	Yes	No
tamoxifen	Yes	No	No
temozolomide	Yes	Yes	No
vinorelbine	Yes	No	No
Total number of significant discrimination	9	6	3

## Foundation model

The concept of transfer learning is instrumental in the development of foundation models, with their effectiveness being significantly amplified by scale [95]. Foundation models are built upon a pretraining approach, aimed at developing a versatile, general-purpose model using extensive datasets and diverse tasks. These models, once created, can be effectively fine-tuned for a variety of specific downstream applications [96]. Trained on a broad array of applications and vast datasets encompassing diverse topics, formats, and languages, foundation models have the capacity to adapt their extensive knowledge to specific requirements efficiently. Their strong adaptation capabilities make them particularly suitable for various tasks in bioinformatics, enhancing both the efficiency and accuracy of applications in this field [97].

In bioinformatics research, foundation models exhibit robust generative capabilities, aiding in tasks such as identifying potential biomarkers, optimizing molecular properties, and designing synthetic biological pathways. These capabilities leverage existing data to accelerate and innovate in the field, as highlighted by [98]. Furthermore, foundation models can integrate various data modalities, enabling a comprehensive exploration of bioinformatics concepts across multiple scales and drawing from an array of knowledge sources [99]. The generative aspects of these models significantly enhance the speed and efficiency of research in areas like drug discovery. They not only reduce time-consuming processes but also assist in the identification of novel and more effective therapeutic compounds [100, 101].

The analysis of downstream tasks in bioinformatics benefits significantly from the application of fine-tuning strategies on foundation models pre-trained with biological knowledge. Li et al. highlight the versatility of foundation models in tackling practical problems within bioinformatics, illustrating how these models offer innovative solutions to the field’s unique challenges [102]. For instance, Liu et al. demonstrate the use of pretraining and contrastive learning on extensive unannotated datasets in an unsupervised manner to accurately identify DNA-binding sites in proteins [103]. Similarly, Yang et al. employ self-supervised learning on large-scale unannotated scRNA-seq data to address batch effects, enhance sequence length, and improve cell type annotation generalizability [104]. Furthermore, Cui et al. utilize gene dimension self-attention mechanisms to optimize performance across various downstream applications, such as gene network inference, cell-type annotation, and multi-omics integration [96]. Foundation models find application in various bioinformatics fields, including biological structure prediction, gene-disease identification, and protein function prediction, showcasing their widespread utility. Beyond the specific downstream analysis tasks mentioned, numerous other applications leveraging foundation models in bioinformatics await further exploration and study.

Large Language Models (LLMs) can be considered a specific subset of foundation models, excelling in various language-related tasks. Models like GPT-4 demonstrate remarkable abilities in question answering and sentence generation [105, 106]. These LLMs exhibit impressive performance in ZSL, enabling them to tackle tasks beyond their initial training scope [107]. They are particularly adept at mitigating OOD issues due to their extensive training on vast and diverse text corpora. This comprehensive training endows them with a profound understanding of language and context, enhancing their generalization capabilities to new, unseen data. LLMs’ size and depth of learned representations facilitate zero-shot transferability to downstream tasks without the need for fine-tuning. They can effectively respond to queries when prompted with task descriptions and examples, showcasing a robust ability to understand context and infer meaning, which is crucial in handling OOD inputs. The application of LLMs in bioinformatics, although in its beginning stages, has shown promising results. Liu et al. highlight the use of LLMs in single-cell data analysis, demonstrating that they can provide stable and reliable results [106]. Additionally, Chen et al. conducted a review evaluating LLMs’ problem-solving abilities in bioinformatics scenarios [108]. They found that while LLMs excel in knowledge acquisition, their proficiency in addressing practical professional queries and conducting nuanced knowledge inference is still evolving.

In summary, LLMs, as a subset of foundation models, have shown significant potential in various fields, including bioinformatics. Their ability to generalize, understand context, and handle diverse data types positions them as valuable tools for addressing OOD issues and beyond.

## Challenges of OOD learning for bioinformatics

In bioinformatics, the advancements in OOD learning have been significant. Traditional machine learning models often struggle with real-world distribution shifts, leading to decreased performance in bioinformatic applications. Employing various OOD learning models effectively addresses the challenges associated with domain shifts. The emergence of OOD learning also fulfills the need for models to adapt to new, unseen data while maintaining accuracy. Furthermore, the integration of multi-omics data, which combines various types of biological data such as genomic, transcriptomic, and proteomic, exemplifies the capacity of OOD learning methods to provide a more comprehensive understanding of biological mechanisms.

However, the field faces significant challenges that must be addressed to fully exploit the potential of OOD learning. Firstly, refining predictive models, particularly in the context of transfer learning, continues to present a significant research challenge. Handling data imbalances and the presence of technical or biological noise pose substantial challenges to accurate OOD prediction [72]. This complexity makes it difficult for models to learn representative features that are robust to OOD samples.

Secondly, a notable issue is the lack of interpretability, which reduces OOD utility in providing meaningful biological insights. This is particularly critical in bioinformatics, where biological validation and understanding are essential for scientific advancement. Additionally, trans-species reliability presents another challenge, particularly in drug response prediction using animal models. Models trained on animal data often fail to generalize to human systems, limiting their translational value and highlighting the need for improved cross-species robustness.

Thirdly, the evolving nature of biological knowledge demands that models continuously adapt to incorporate new, often OOD, information. Real-time adaptability is critical to maintaining relevance in rapidly advancing fields.

Fourthly, the characteristic multi-modality of biological data also presents challenges. The potential dissimilarities between pre-clinical models and human tumors, which might also be present in other molecular data types, present additional challenges. This multi-modality often results in inconsistent integration of diverse data types, reducing model effectiveness. Furthermore, the inability to account for uncertainties across these modalities exacerbates the problem. Machine learning models often lack robust uncertainty quantification mechanisms, making it difficult to evaluate the reliability of predictions derived from multi-modal datasets. This limitation is particularly concerning in scenarios requiring precise cross-modal integration, as uncertainty in one modality can propagate and undermine overall model performance.

Finally, the lack of standardized benchmarks for evaluating OOD samples remains a significant obstacle. The limitation impedes the development and comparison of robust OOD learning models specially designed to handle OOD samples, as opposed to models optimized for ID scenarios [109]. Establishing standardized evaluation frameworks is essential for advancing robust OOD learning methodologies in bioinformatics.

## Outlook of OOD learning for bioinformatics

The outlook for OOD learning in bioinformatics is highly promising and has the potential to be transformative, given the field’s unique challenges and the crucial need for robust and generalizable models. Bioinformatics spans an extensive range of data types, including genomic sequences, protein structures, and metabolic pathways, each characterized by distinct patterns and distribution shifts. OOD learning is poised to significantly improve how models process, interpret, and make predictions on this diverse and frequently novel data. To address some challenges outlined and provide some insights for future research, several directions and strategies are proposed.

Firstly, advancing OOD learning requires the refinement of TL techniques. Emerging methods such as Low-Rank Adaptation (LoRA) and prompt learning offer promising solutions for enhancing the efficiency and accessibility of TL [110]. LoRA is a method that reduces the computational and storage requirements of fine-tuning large models by decomposing the weight updates into low-rank matrices. These innovations enable parameter transfer with minimal computational cost, making TL feasible for resource-constrained domains. By fine-tuning only a small portion of a pretrained model and employing prompts to guide task adaptation, prompt learning facilitates effective use of pretrained models while minimizing reliance on extensive labeled datasets and large task-specific data requirements. Together, these techniques hold the potential to make TL more efficient and broadly applicable across diverse bioinformatics fields.

Secondly, addressing temporal shifts in DA and DG and incorporating time-series analysis alongside statistical alignment could enhance adaptability to evolving datasets. Future efforts should focus on incorporating time-independent features to better adapt to gradual changes in data distribution [111]. Combining DA and DG with time series analysis can effectively address both feature and label shifts, enhancing overall performance [112]. Additionally, current DA or DG methods in bioinformatics often prioritize statistical alignment while overlooking underlying causal relationships. Leveraging causal graphs or structural causal models could provide a more robust framework for guiding adaptation and interpretability.

Thirdly, representation learning in multimodal data poses a significant transformation. To thrive in OOD scenarios, models need to extract features that are invariant across domains and modalities while remaining distinctive within classes. Therefore, extracting features with low intra-class variation and high inter-class separation is crucial for effectively handling OOD data. Developing improved representation methods can significantly enhance OOD learning in bioinformatics. Specifically, promoting domain-invariant representations in hyperspherical space has emerged as a promising strategy [113]. Such representations maintain consistency across domains and modalities while preserving the distinctiveness of class-level features, fostering robust generalization in complex biological datasets.

Fourthly, OOD learning models that can adapt in real time to the ever-changing landscape are also critical. Continual learning is particularly beneficial in the dynamic field of bioinformatics, where new discoveries and datasets emerge at a rapid pace. By enabling models to adapt continuously to new information, continual learning ensures that they remain relevant and effective in addressing evolving biological questions. Implementing dynamic models capable of continual learning allows systems to incorporate novel insights and datasets seamlessly, without the need for retraining from scratch [114].

Fifthly, beyond adaptability, the growing availability of computational resources is opening new doors for OOD learning. Foundation models, such as GPT-4 and BioBERT, haven demonstrated exceptional performance in bioinformatics, even with OOD data [115]. Future efforts in bioinformatics could focus on training foundation models using cohort-specific datasets, such as the UK Biobank, and transferring the knowledge to different cohorts with similar data types but differing distributions. Additionally, leveraging cross-modal inference could enrich or impute missing information in one modality using data from another, enabling the fusion of multi-modal information for improved downstream analyses [116]. This approach holds the potential to enhance our understanding of underrepresented cohorts, ultimately contributing to more equitable and robust bioinformatics insights.

Finally, addressing the variability in model performance through uncertainty quantification is essential. Conformal inference is a statistical framework that generates prediction intervals with a guaranteed error rate, regardless of the underlying data distribution [117, 118]. An important area for further research is how to obtain distribution-free prediction intervals for a target population, particularly under distribution shifts and while integrating multiple data sources [119]. This approach could enhance the robustness and reliability of predictions, even in the presence of complex and heterogeneous data distributions.

In summary, while OOD learning has brought significant advancements in bioinformatics, enhancing the understanding of complex biological data and improving predictive modeling, several challenges persist. Addressing these challenges will not only solidify the role of OOD learning in bioinformatics but also open new avenues for scientific discovery and innovation. As the field continues to evolve, OOD learning offers a pathway toward a deeper understanding of biological systems and improved outcomes in healthcare and biomedical research.

**Key Points**

- OOD learning is crucial for addressing distribution shifts in bioinformatics, allowing models to maintain performance on unseen data.
- OOD learning is applied across diverse fields, demonstrating its versatility in solving real-world biomedical challenges.
- OOD learning faces ongoing challenges that must be addressed to further enhance bioinformatics, healthcare, and research applications.

## Supplementary Material

Supplementary_materials_Table_S1_bbaf294

Supplementary_materials_Table_S2_bbaf294

Supplementary_materials_Table_S3_bbaf294

## Data Availability

Data sharing is not applicable to this article as no datasets were generated or analysed during the current study.
